# Mental workload during n-back task—quantified in the prefrontal cortex using fNIRS

**DOI:** 10.3389/fnhum.2013.00935

**Published:** 2014-01-16

**Authors:** Christian Herff, Dominic Heger, Ole Fortmann, Johannes Hennrich, Felix Putze, Tanja Schultz

**Affiliations:** Cognitive Systems Lab, Institute for Anthropomatics, Karlsruhe Institute of TechnologyKarlsruhe, Germany

**Keywords:** fNIRS, near-infrared spectroscopy, prefrontal cortex, workload, mental states, user state monitoring, n-back, passive BCI

## Abstract

When interacting with technical systems, users experience mental workload. Particularly in multitasking scenarios (e.g., interacting with the car navigation system while driving) it is desired to not distract the users from their primary task. For such purposes, human-machine interfaces (HCIs) are desirable which continuously monitor the users' workload and dynamically adapt the behavior of the interface to the measured workload. While memory tasks have been shown to elicit hemodynamic responses in the brain when averaging over multiple trials, a robust single trial classification is a crucial prerequisite for the purpose of dynamically adapting HCIs to the workload of its user. The prefrontal cortex (PFC) plays an important role in the processing of memory and the associated workload. In this study of 10 subjects, we used functional Near-Infrared Spectroscopy (fNIRS), a non-invasive imaging modality, to sample workload activity in the PFC. The results show up to 78% accuracy for single-trial discrimination of three levels of workload from each other. We use an *n*-back task (*n* ∈ {1, 2, 3}) to induce different levels of workload, forcing subjects to continuously remember the last one, two, or three of rapidly changing items. Our experimental results show that measuring hemodynamic responses in the PFC with fNIRS, can be used to robustly quantify and classify mental workload. Single trial analysis is still a young field that suffers from a general lack of standards. To increase comparability of fNIRS methods and results, the data corpus for this study is made available online.

## 1. Introduction

Functional Near-Infrared Spectroscopy (fNIRS) is an imaging modality measuring hemodynamic processes in the brain. It provides insights into the same activation patterns as functional Magnetic Resonance Imaging (fMRI), the de facto standard in neuroscience research, while not confining the subject in a small space. Thereby, it allows for measurements of large subject populations outside of clinical environments. Besides montages covering the whole head, fNIRS sources and detector optodes can also be placed on the subjects head to measure exactly the parts of the cortex that contain relevant activations for the investigated task. When the region of interest is known beforehand, this can be used to design optode holders that can be fixed in place in less than 1 min. Potentially, fNIRS could thus be used in real world scenarios, as well.

Most fNIRS studies investigate differences in average activation patterns for different conditions. Only very recently has fNIRS been used to classify single-trial activations for Brain-Computer Interfacing (Coyle et al., 2007). A Brain-Computer Interface is a communication channel between the brain and a computer through interpretation of neural activation pattern (Wolpaw et al., 2002). Nearly all existing single-trial studies differentiate fNIRS patterns of subjects performing a cognitive task from the rest state or no-control state. The most frequently used paradigm is motor-imagery (Sitaram et al., 2007).

Recently, neural signals have been used to adapt and complement traditional input sources, such as keyboard and mouse, by adapting the interface to the users' state instead of directly controlling the interface. These so called passive Brain-Computer Interfaces (Cutrell and Tan, 2008; Zander and Kothe, 2011) mostly use the Electroencephalogram (EEG). Passive Brain-Computer Interfaces (BCIs) often measure a user's state and adapt a user interface accordingly. In fNIRS, multiple studies investigate mental arithmetics (Ang et al., 2010a) to monitor users' engagement in arithmetic tasks. Power et al. (2012) investigate the consistency of mental arithmetic classification across different sessions. Instead of recognizing mental arithmetics, Power et al. (2010) show that mental arithmetic and music imagery lead to distinct activation patterns that can be classified in single trial analysis. Following up on this idea, Herff et al. (2013) differentiate three different mental tasks, namely mental arithmetics, mental rotation and word generation. Girouard et al. (2009) distinguish between two difficulty levels in the popular game Pac-Man, instead of discriminating from a rest state. Ang et al. (2010b) show robust classification for three difficulty levels in mental arithmetics using fNIRS to evaluate numerical cognition class-room settings. While Ang et al. focus on the differentiation of difficulty levels, our focus is on the classification of mental workload induced by a memory task. Recently, Hirshfield et al. (2011) evaluated the type of cognitive demand placed on a user by different types of tasks. The focus of their study is on the type of workload, while we are aiming at the quantification of workload in this study.

In a multi-modal study using blood volume pressure, respiration measures, electrodermal activity and EEG, Jarvis et al. (2011) measured workload in a driving simulator to adapt a driving assistant. Workload has been of interest in the fNIRS community, as well. Cognitive workload has been assessed for air-traffic controllers in several studies Ayaz et al. (2010, 2012). Izzetoglu et al. (2003) show that task load in the Warship Commander tasks yield distinct hemodynamic responses on average. Aiming at a usage for BCI, Ayaz et al. (2007) analyze workload induced by the *n*-back tasks, but limit their results to grand averages, as well. However, these studies look at average hemodynamic responses and do not attempt single trial analysis. To use these findings to adapt interfaces to the user's current workload, the hemodynamic responses have to be analyzed in single trial. Proving that a cognitive task yiels hemodynamic responses on average does not automatically mean that the activations can be robustly recognized in single trial, which is necessary if interfaces should be adapted. In this work, we provide evidence that different levels of workload yield hemodynamic responses that can be robustly classified without averaging.

Findings in EEG Brouwer et al. (2012); Berka et al. (2007) show that workload induced by the *n*-back task can be classified in single trial. Baldwin and Penaranda (2012) demonstrate how the models trained on one workload condition can be transferred to others in EEG. In this study, we show that the workload induced by different *n*-back conditions results in hemodynamic responses that are consistent enough to be classified on a single trial basis. We use an *n*-back task to induce different levels of workload, forcing subjects to continuously remember the last one, two, or three of rapidly changing items. To enable realistic passive BCIs, we not only evaluate whether a user is engaged in a task, but quantify the level of mental workload the user experiences during the *n*-back task (*n* ∈ {1, 2, 3}). Thereby, we quantify workload using fNIRS.

In functional imaging studies, the prefrontal cortex (PFC) has been identified to be among the relevant areas for memory related tasks (Smith and Jonides, 1997). The PFC has been found to be relevant both in PET (Smith and Jonides, 1997) and fMRI studies (Cohen et al., 1997). An in depth meta-analysis of *n*-back studies using fMRI (Owen et al., 2005) confirms the importance of the PFC for *n*-back. Hoshi et al. (2003) show spatio temporal changes for working memory tasks in the PFC using fNIRS. Their analysis is based on averages and does not include single trial analysis, but confirms that fNIRS is ideally suited for measurements of the PFC. An fNIRS headset can be quickly fixed to the forehead and enables measurements of the PFC within minutes, while guaranteeing high data quality. In an investigation using finger tapping and fNIRS, Cui et al. (2010b) show that the delay in fNIRS-based BCIs can be reduced to further improve the usability of fNIRS in real-life scenarios. Workload induced by a memory task and fNIRS-based measurement of the PFC are thus an ideal combination for a realistic passive BCI to monitor workload levels.

## 2. Materials and methods

### 2.1. *n*-back

In the *n*-back task, users have to continuously remember the last *n* of a series of rapidly flashing letters. The *n*-back task requires subjects to react when a stimulus is the same as the *n*-th letter before the stimulus letter. We denote a (letter) stimulus, which is the same as the one *n* previously as a target. Subjects had to press the space key on a keyboard when they encountered a target. With increasing *n* the task difficulty increases, as the subjects have to remember more letters and continuously shift the remembered sequence. Performance in this task can be evaluated by measuring the amount of missed targets, when the subjects do not press the key for a target and through the amount of wrong reactions, when the subjects incorrectly identify a stimulus letter as a target.

### 2.2 NIRS data recording

Like fMRI, fNIRS measures changes in blood oxygenation in brain areas triggered by neural activity. Using light in the near-infrared range of the electromagnetic spectrum (620–1000 nm), which disperses through most biological tissue but is absorbed by hemoglobin, the level of oxygenated and deoxygenated hemoglobin (*HbO* and *HbR*) can be estimated using the modified Beer-Lambert law (Sassaroli and Fantini, 2004).

We used an Oxymon Mark III by Artinis Medical Systems to measure fNIRS signals. The system uses two wavelength of 765 and 856 nm and outputs concentration changes of *HbO* and *HbR*. To measure hemodynamic activity in the PFC, we attached four transmitter and four receiver optodes to the forehead. Each detector measures time-multiplexed from two sources, located at a distance of 3.5 cm, resulting in a total of 8 channels of *HbO* and *HbR*. Our signals were sampled at 25 Hz.

**Figure 1:**
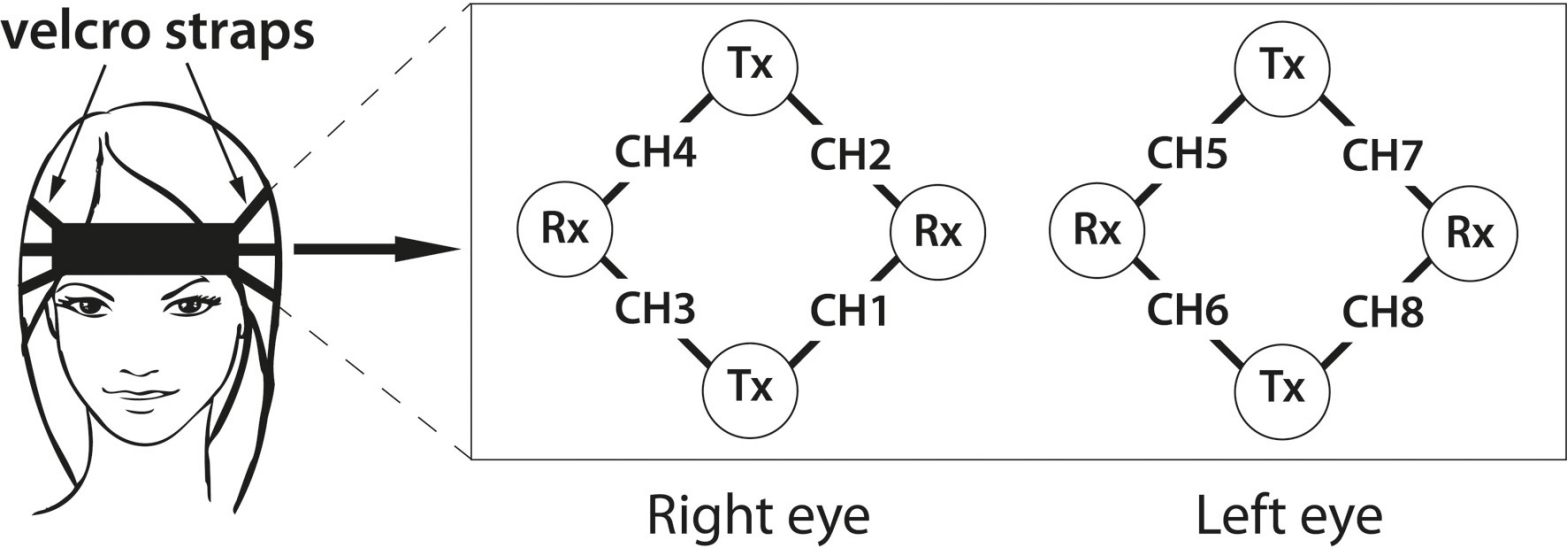
**Optode placement in our experiment**. Transmitter optodes are marked as Tx, while Rx indicates receiver optode positions.

Figure 1 shows the placement of our optodes on the subjects' forehead. The recording setup on the forehead is very simple and needs less than 3 min to be fixed in place and to assess data quality.

### 2.3. Experiment design

In our experiment, we investigated 10 trials each of 1-,2-, and 3-back tasks. Each trial contained 3 ± 1 targets. The experiment was presented to the subjects on a screen, which was placed in front of them in 50 cm distance.

**Figure 2:**
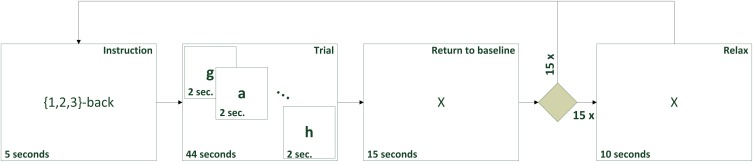
**Experimental design for *n*-back task**.

A trial consisted of 5 s of instruction, informing the subject which task (1-,2- or 3-back) was about to start. The trial then presented a new letter every 2 s. Every letter was displayed for 500 ms. The screen was left blank for the remaining 1.5 s. A total of 22 letters was presented during every trial resulting in a trial length of 44 s. Subsequently, a cross was displayed for 15 s during which the subjects were asked to relax to ensure that hemoglobin levels returned to baseline. We excluded these periods from our analysis, as they are strongly influenced by the previous hemodynamic responses. After half of the trials, an additional 10 s of the resting cross were displayed to have data periods with no activity to be used as relax trials. We intentionally use periods with true relax signals for our analysis instead of periods in which *HbO* and *HbR* returned to baseline. Figure 2 shows the experiment protocol. The order of the different *n*-back conditions was pseudo-randomized. A 150 s break during which the subjects could drink or chat was included after 15 trials. The entire experiment had a recording time of 37 min (30 trials of 64 s, 15 relax trials of 10 s and 150 s in the middle).

The fNIRS data was recorded continuously during the entire session. The trials were segmented afterwards based on the time sequence induced by the described experimental setup. In addition to the recorded fNIRS data, subjects filled out a questionnaire regarding their age, occupation, handedness and a series of questions about the experiment on a 6-point Likert scale. The scale ranged from “no agreement” (1) to “complete agreement” (6) for a given statement. We asked our subjects how much they agreed with the statements “The *n*-back task was demanding,” to evaluate subjective workload. Subjects were asked to judge their level of concentration during the first and second half of the experiment by indicating their agreement with the statement “I was very concentrated.” Additionally, subjects indicated their agreement with the phrase “The system is comfortable to wear.” Lastly, we evaluated whether our participants thought that the duration of the experiment was appropriate. Section 3.1 contains results of the questionnaire evaluation.

### 2.4. Participants

In this study, we recorded 10 subjects (4 females) with a mean age of 22 years. Using the Edinburgh handedness inventory Oldfield (1971), we evaluated the handedness of our subjects. In total, we had 8 right-handed and 2 left-handed participants. All subjects had normal or corrected to normal vision. The participants were informed prior to the experiment and gave written consent. None of the subjects had ever taken part in an *n*-back study before to ensure that no training effects are present.

To increase comparability between fNIRS methods and results, the complete data collected in this study will be shared with the community (see Section 4.1).

### 2.5. Signal processing and artifact removal

The signals measured by fNIRS are subject to biological and technical artifacts. Cardiovascular effects like heart-beat, respiration and slow waves (e.g., Mayer Waves) influence the recorded data. Movement artifacts which alter the position of the optodes and lift them off the scalp, causing spikes in the recordings, are present in most fNIRS datasets, as well. A general overview of fNIRS artifacts and artifact removal techniques can be found in Cooper et al. (2012).

To attenuate trends and Mayer Wave like effects, we used a moving average filter, which subtracted the mean of the 120 s before and after every sample from every *HbO* and *HbR* datapoint. Moving average filters have been used successfully before to remove slow trends in experiments with long trials (Heger et al., 2013). Heart-beat and faster frequency signals are attenuate using an elliptical IIR low-pass filter with cutoff frequency of 0.5 Hz and filter order of 6, which robustly reduces heart-beat influences in the data. Finally, we used a wavelet artifact removal method (Molavi and Dumont, 2010) to reduce the effect of movement artifacts.

The trials were then extracted based on the experiment timings and associated with a label according to the *n*-back condition or relax. Each trial of any of the *n*-back conditions is 44 s long, while the relax trials are 10 s long.

### 2.6. Feature extraction and selection

Typical hemodynamic responses increase for *HbO* with neural activity in a specific region and return to baseline afterward. In *HbR*, signals typically behave opposite and decrease upon stimulus onset and increase back to baseline after the end of the stimulus. This typical behavior is often used in the feature extraction. The mean value of the signal (Heger et al., 2013) in a specific window or the increase in mean value between different windows (Herff et al., 2012) is often used as a simple, but effective feature. In this study, we use the slope of a straight line fitted to the data in a window as the feature. The line was fitted using linear regression with a least-square approach. Window sizes were varied in the experiments. Even though *HbO* and *HbR* signals of every channel are strongly negatively correlated (Cui et al., 2010a), we extract the slope feature for *HbO* and *HbR* of every channel. Including both *HbO* and *HbR* signals often yields more robust classification results. This results in 16 features per window, as we extract one feature for *HbO* and one for *HbR* for each of the 8 channels.

To reduce the feature set size, we only include features with a high relevance for classification in the feature set. We calculate the Mutual Information between each continuous feature and the discrete labels on the training data using non-parametric probability density functions. These were estimated using kernel methods (Parzen windows). See Ang et al. (2008) for a more detailed description of feature selection methods using Mutual Information. In this study, we limit our feature set to the 8 features containing the highest Mutual Information with the labels, as the remaining half of the features only contained little to no relevance.

### 2.7. Evaluation

To classify the data, we used a Linear Discriminant Analysis (LDA) classifier. For the multi-class experiments, we used a one-vs-one multi-class classifying approach (Duda et al., 2012). To evaluate classification accuracy in our experiment, we used a 10-fold cross-validation. For this, the data of one subject is divided into 10 equally sized parts and in a round-robin manner, 9 parts are used for feature selection and training, while the last part is used for evaluation. Presented accuracies are then averaged over all 10 folds. We only evaluate subject dependend systems in this paper. As we use a 10-fold approach and have 10 trials per class, we never use any data shortly before or after the testing data, which could be problematic given the high auto-correlation of fNIRS signals. To evaluate our data set, we first classified the three *n*-back classes from relax. The relax trials are only 10 s long, while the *n*-back trials last 44 s. We only extracted 10 s long windows from *n*-back classes for this task, as well. Therefore, we evaluated the effect on classification accuracy resulting from different offsets from the start of a trial.

To really quantify mental workload we evaluate classification between the three *n*-back classes. We evaluate classification accuracy depending on window length in which we extract the slope feature.

## 3. Results

### 3.1. User performance and subjective rating

**Figure 3:**
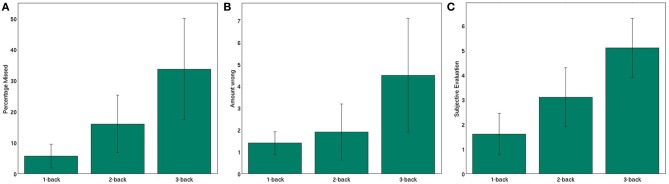
**User performance and subjective evaluation in the *n*-back task (A) average number of missed targets (B) average number of wrong reactions (C) average subjective evaluation of task difficulty**. Whiskers show standard deviations between subjects. All differences between the conditions are significant (tested by one-sided *t*-tests, *p* < 0.05 after Bonferroni correction), except for the difference between 1 and 2-back in **(B)**.

To confirm that our subjects perceived the different *n*-back conditions as different, we analyzed the user performance. Figure 3 shows user performance and subjective evaluation of the experiment.

We evaluated the amount of missed targets, when a subject failed to press the key when a target stimulus was presented. A One-Way ANOVA shows significant differences between the three *n*-back levels in the amount of missed targets (*F* = 16.3151; *p* < 0.001). The percentage of targets missed by the subjects increased from 5.7% on average for the 1-back condition to 16.7% for 2-back to 33.7% for the 3-back task. This clearly shows that the three tasks have significantly different difficulty levels (tested by one-sided t-tests, *p* < 0.01 after Bonferroni correction all three comparisons). Additionally, this clarifies that even in the 3-back tasks our subjects identified two thirds of the targets. Next, we evaluated the amount of wrong reactions, when subjects incorrectly identified a letter as a target and pressed the space key. The amount of wrong reactions is significantly influenced by the *n*-back level (tested by ANOVA, *F* = 9.613; *p* < 0.001). Again, the number of wrong reactions increases from 1.4 on average to 1.9 to 4.5. The differences in wrong reactions between 1 and 3-back and 2 and 3-back are significant (tested by one-sided t-test *p* < 0.01 after Bonferrroni correction), while the difference between 1 and 2-back is not statistically significant. The subjective evaluation of the subjects agreeing with the phrase “The *n*-back task was demanding,” clearly shows the different mental workload levels of the three conditions (statistically significant as tested by One-Way ANOVA, *F* = 25.8540; *p* < 0.001). While the average agreement was 1.6 (1 meaning no agreement) for 1-back, subjects answered 3.1 for 2-back and 5.1 on average for 3-back (6 being total agreement). All differences between the three classes are significant (tested by one-sided t-tests *p* < 0.01 after Bonferroni correction). This clearly shows the different levels of workload induced by the three *n*-back conditions.

Subjects stated that they were highly concentrated during the first half of the experiment, answering that they agreed with 4.9 with the phrase “I was concentrated during this half of the experiment.” This decreased slightly to 4.0 for the second half. The fNIRS system was judged as being comfortable to wear (3.9 in agreement to a comfortable system) in the first half, which decreased to a medium 2.7 for the second half. Our subjects evaluated the duration of the experiment as appropriate (agreement of 4.7).

### 3.2. Hemodynamic responses

**Figure 4:**
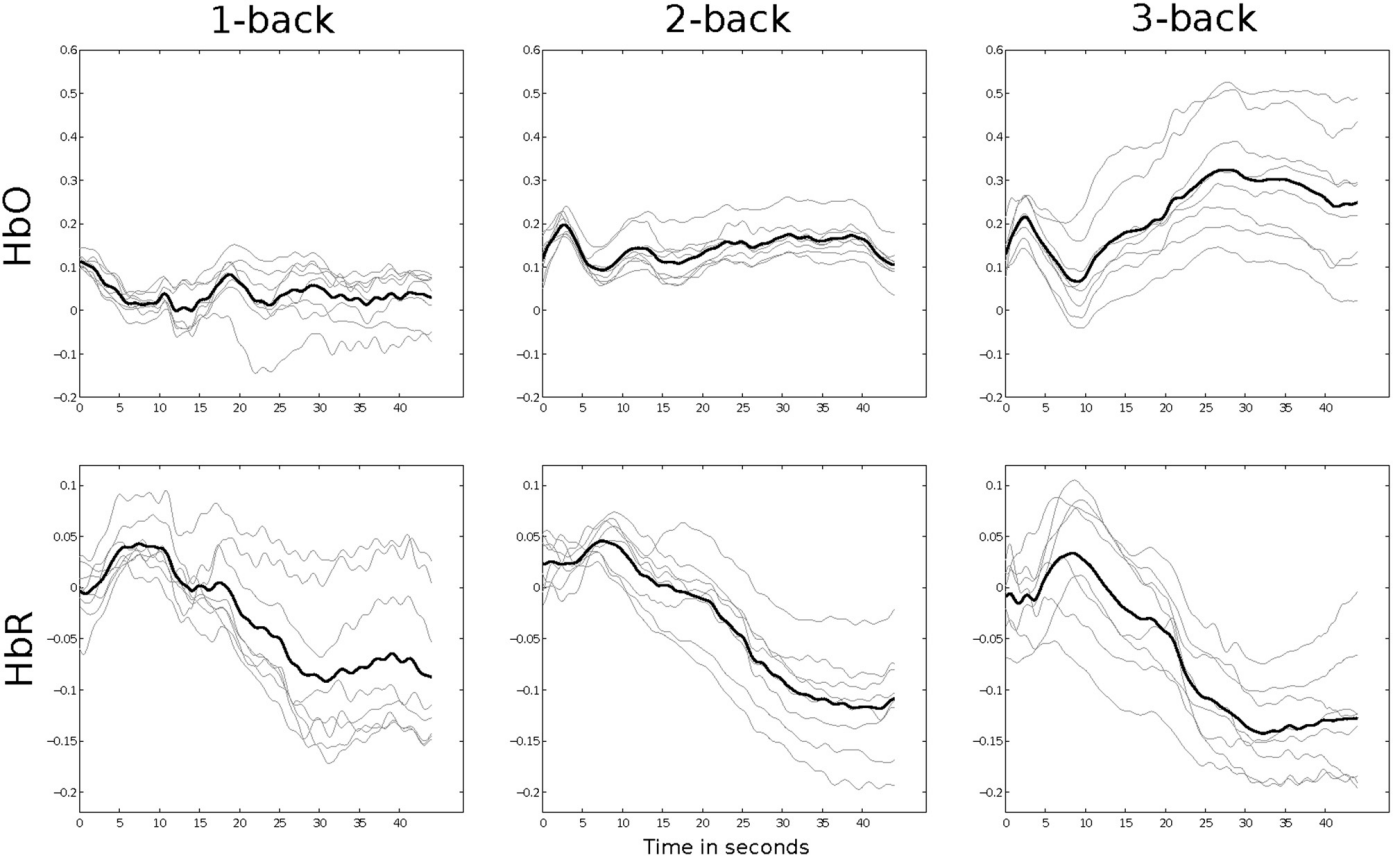
**Grand averages of all 10 subjects in the three *n*-back conditions**. Gray lines indicate single channels. The black line presents the mean of all channels.

To see whether the Hemodynamic responses for the three *n*-back conditions yield any differences, we first analyze the grand averages of all subjects. For this analysis, we baseline every trial by subtracting the mean of the 10 s prior to the trial for *HbO* and *HbR* of every channel. The trials are not baseline normalized for the remaining classification analyses. Figure 4 shows grand averages for all channels and all *n*-back conditions.

Gray lines show grand averages for individual channels, while the black line shows the mean over all channels. In the *HbO* channels, there is little activity for 1- and 2-back, but a clear increase for most channels in the 3-back conditions. It is obvious that a feature derived from the slope of those grand averages could discriminate the 3-back trials from the others. In *HbR* the typical decrease can be seen for all three conditions. While the slope is negative for all three tasks, it is clearly steeper in the 2-back grand average than in the 1-back and steepest for the 3-back averages. These grand averages show that we have different activation patterns for the three conditions and visualize the basis of our classification.

### 3.3. *n*-back vs. relax

**Figure 5:**
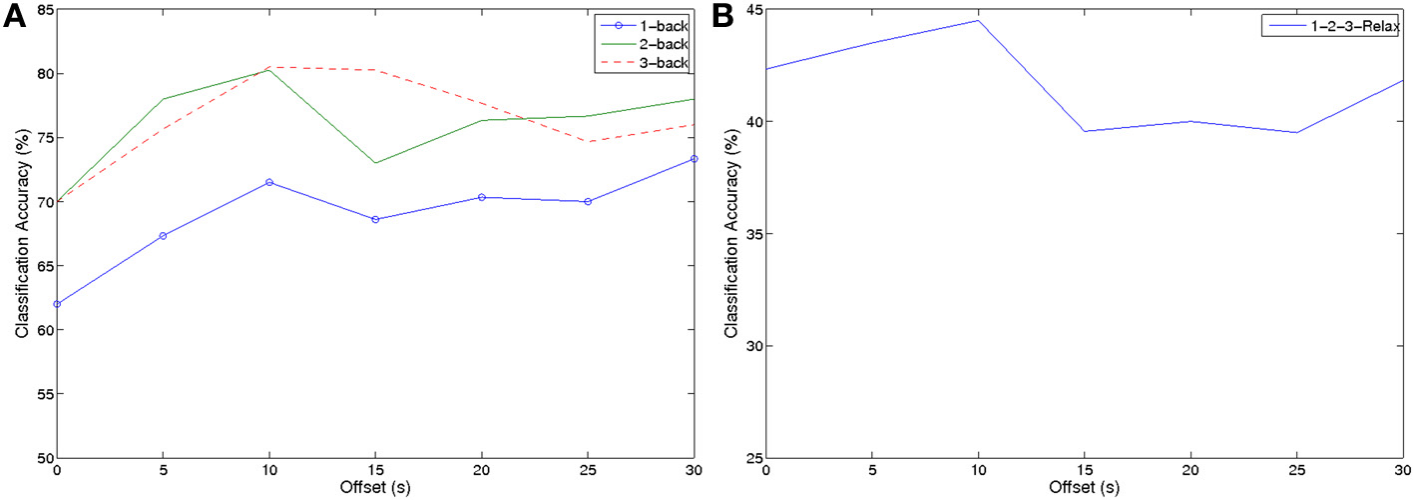
**Classification accuracies for *n*-back tasks depending on the offset from trial start (A) two class problems of classification accuracy of 1-,2-,3-back against Relax (B) four class classification between all three *n*-back and Relax**.

To evaluate the data set we first classified our *n*-back trials from the relax trials collected after the signals returned to baseline. Since our relax trials are only 10 s long, while our *n*-back trials are 44 s in length, we evaluated the effect the offset from the beginning of the trial has on classification accuracies. Figure 5 shows the classification accuracies depending on the offset from the beginning of the trial when extracting the 10 s long windows.

Extracting the 10 s long window directly after the beginning of the trial yields the worst results for all conditions. This can be explained by the fact that subjects are only beginning to memorize the stimuli and are not experiencing workload yet. After an offset of 10 s the results remain relatively stable. All results are significantly better than chance level (tested by Wilcoxon rank-sum). Even in the four-class classification task we could achieve accuracies up to 45% (chance 25%). As expected, classifying 3-back against Relax yielded the best results of up to 81% accuracy. For 2-back, we could achieve 80% accuracy for classification against Relax and 72% for 1-back, respectively. These results show that the single trial data can be robustly discriminated from a relax state.

Table 1 summarizes classification accuracies of each of the conditions against relax and for the four class experiment with an offset of 10 s. These results can be used to compare with previous studies which focus on discriminating from the relax state.

**Table 1:** **Classification accuracies of the conditions against a relax state**.

	**1-back**	**2-back**	**3-back**	**1-2-3-relax**
Mean	71.5%	80.3%	80.5%	44.5%
Standard deviation	17.7	10.5	13.8	10.0
Chance level	50%	50%	50%	25%

### 3.4. Quantifying mental workload

**Figure 6:**
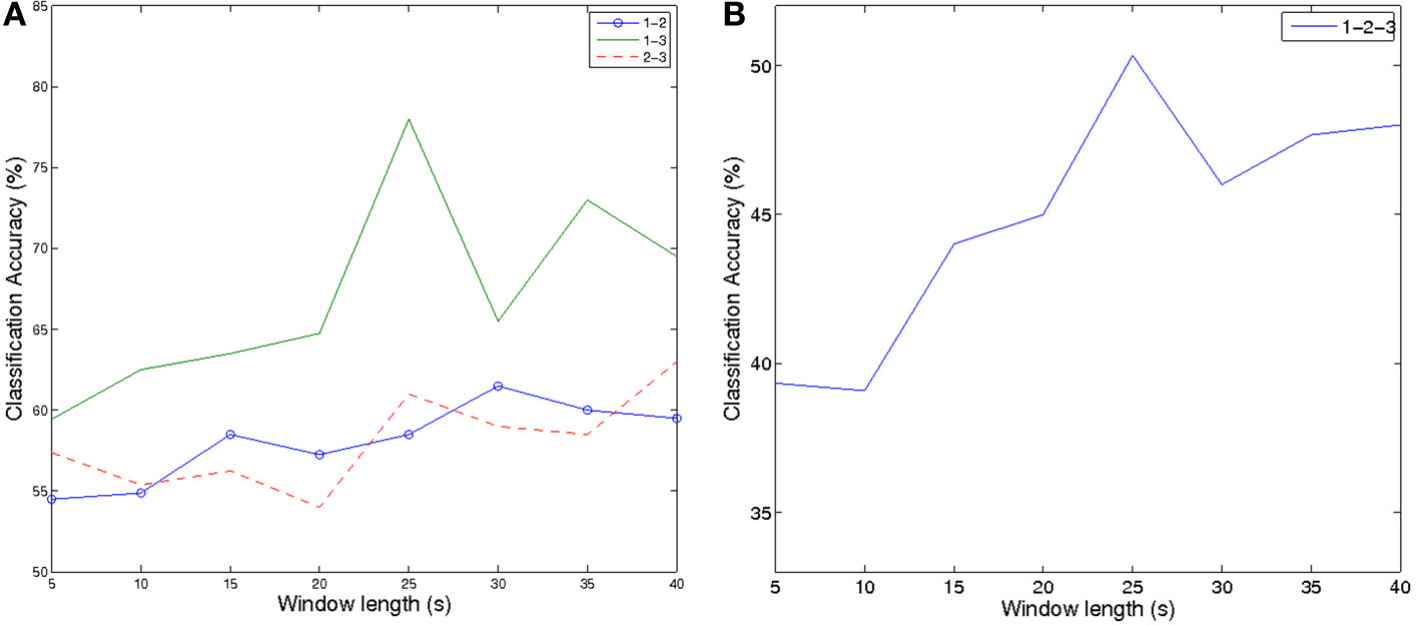
**Classification accuracies depending on window length (A) two class problems between different workload levels (B) three class classification of all three workload levels**.

To quantify workload it is necessary to discriminate different levels of workload from each other and not only from a relax state. We investigate the three *n*-back conditions against each other in two class and three class scenarios. To evaluate the window length necessary for robust classification of mental workload, we show classification accuracies depending on window length in Figure 6.

Part **(A)** of Figure 6, shows accuracies for the two class discrimination between two levels of workload, while part **(B)** shows the three class accuracies of all three workload levels. Note that with increasing window size, the amount of instances reduces. While we can extract 80 instances for a window length of 5 s, this amount reduces to 10 for window lengths larger than 25 s. The little amount of training and testing data sets explains the unstable results for window lengths longer than 25 s.

**Figure 7:**
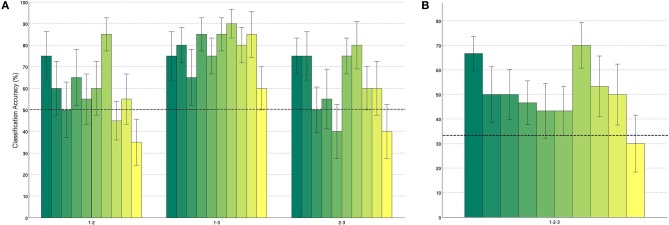
**Classification accuracies for each subject with window length of 25 s (A) two class problems (B) three class classification**. Each bar represents classification accuracies of one subject. The dotted line denotes naive classification accuracy. Whiskers show standard error in the cross-validation.

Results increase for increasing window lengths and peak for the length of 25 s. The discrimination between 1- and 3-back works best, which can easily be explained as the degree of difficulty is most different in those two conditions. Classification between 1- and 2-back and 3- and 2-back yield comparable results as the difference in difficulty level across these conditions is similar. For longer window lengths, these results are significantly better than chance level. The three class experiment is above chance for all window lengths and peaks at 50% accuracy for 25 s window length. The detailed results for every subject for window length of 25 s can be found in Figure 7. It can be seen that all subjects yield good results for the discrimination between 1-3 back, while only roughly half of the subjects work well for the other two scenarios. The results across subjects are significantly better than chance level for all classification scenarios (tested by Wilcoxon rank-sum tests).

Table 2 summarizes the mean results across all subjects for window lengths of 25 s and 15 s. We present the results for window length of 15 s as well, as this length has been used for workload evaluation with EEG before (Kothe and Makeig, 2011). The results for 25 s long windows clearly show that fNIRS signals can be used to robustly quantify different levels of workload. This is a large step toward passive BCIs using fNIRS for workload monitoring.

**Table 2:** **Classification accuracies of the conditions against each other**.

**Window length**	**1-2**	**1-3**	**2-3**	**1-2-3**
15 s	58.5%	63.5%	56.3 %	44.0%
25 s	58.5%	78.0%	61.0%	50.3%
Chance level	50%	50%	50%	33.3%

## 4. Discussion

In this study of 10 subjects, we show that fNIRS signals measured from the PFC with an easy to setup montage can be used to robustly quantify users' workload. The analysis of user performance show significant differences in the amount of missed targets and wrong reactions depending of the *n*-back level. Additionally, the subjective evaluation of the users show big differences in perceived difficulty level between the *n*-back levels, as well.

Using 8 channels on the forehead, we were able to classify the different levels of workload induced by *n*-back tasks from a relax state with accuracies up to 81%. As expected, 3-back could be discriminated best from the relax state (81% accuracy), as the mental workload induced by this condition is the largest. However, classification of 2-back and 1-back against relax still yielded mean accuracies of 80 and 72%, respectively. These results show that even the workload induced by relatively simple tasks can be robustly discriminated from a resting state.

More importantly, the hemodynamic responses measured in the PFC are consistent enough to be used to discriminate between three levels of workload. While the classification of high vs. low workload (1 vs. 3-back) worked well for all 10 subjects and yielded an average of 78% accuracy, the discrimination between 1 and 2-back only resulted in usable results for half of the subjects (average of 58.5%). Classification between the workload induced by 2 and 3-back tasks resulted in an average of 61% accuracy. These results mirror the subjective and user performance evaluation, as the difference between 1 and 3-back is largest and the difference in workload induced by 1 and 2-back seems to be smallest (no significant difference in the amount of errors between those two conditions).

We thereby show the potential of fNIRS as a modality for passive BCI and user state monitoring, despite the fact that further investigation is necessary to differentiate between more levels of workload with higher accuracies. The simple optode montage and the robust results encourage fNIRS to be used in real-life scenarios like car navigation and class-room settings. In this study, the data was analyzed in an offline manner and especially the moving average filter needs to be adapted for usage in an online system. Instead of only classifying whether a subject was engaged in a task or not, we were able to reliably show the degree of workload a subject was experiencing. The presented results thus show the feasibility of using fNIRS to quantify workload in single trial.

### 4.1. Data sharing

Single-trial analysis of fNIRS data is still a very young field and to the best of our knowledge, there are only very few publicly available data sets of single trial fNIRS experiments. To increase comparability of single trial fNIRS methods and allow for benchmarking, the data corpus used in this study will be publicly available on the authors' website^1^. The fNIRS time courses for all 10 subjects and for all *n*-back conditions and Relax can be downloaded in both MATLAB™ and Comma-Separated-Value (CSV) file formats. The questionnaire and behavior results will be included, as well. Thereby, we hope to provide a common data set for evaluation and testing of fNIRS methods and algorithms.

### Conflict of interest statement

The authors declare that the research was conducted in the absence of any commercial or financial relationships that could be construed as a potential conflict of interest.

## Footnotes

^1^http://csl.anthropomatik.kit.edu/english/2506.php
